# Temporary meniscus extrusion is caused by cumulative stress from uphill and downhill tasks in healthy volunteers

**DOI:** 10.3389/fspor.2024.1271987

**Published:** 2024-04-08

**Authors:** Yosuke Ishii, Saeko Okamoto, Riko Okinaka, Takato Hashizume, Chen Xu, Kexin Zhu, Yuko Nakashima, Kaoru Okada, Kazuya Takagi, Nobuo Adachi, Makoto Takahashi

**Affiliations:** ^1^ Department of Biomechanics, Graduate School of Biomedical and Health Sciences, Hiroshima University, Hiroshima, Japan; ^2^ Department of Orthopedic Surgery, Graduate School of Biomedical and Health Sciences, Hiroshima University, Hiroshima, Japan; ^3^ Ultrasound Business Operations, Healthcare Business Headquarters, Konica Minolta, Inc., Tokyo, Japan

**Keywords:** meniscus extrusion, ultrasonography, cumulative load, uphill/downhill loading, healthy individual

## Abstract

**Purpose:**

Excessive mechanical stress accumulates and causes knee injury. Meniscal extrusion is a key factor in detecting the reaction to cumulative mechanical stress. The accumulation of stress strongly depends on environmental conditions such as flat ground or uphill/downhill, and accumulates in knee compartments; only a few studies have reported the effects of different environments on lateral and medial meniscus extrusion. This study aimed to investigate the effects of cumulative uphill/downhill stress on the meniscal extrusion in each compartment.

**Methods:**

A total of 30 healthy volunteers with 30 affected knees were involved in this cohort study (mean age, 22.0 ± 1.1 years; men, *n* = 14). The participants were divided into flat-walking, uphill/downhill-walking, and uphill/downhill-jogging groups and their numbers of steps taken were recorded during the effort. Moreover, medial and lateral meniscal extrusions during walking were evaluated using ultrasound three times, before and after efforts (T1) and (T2), and one day after efforts (T3), respectively.

**Results:**

In the flat-walking group, no significant differences were observed between the follow-up periods. Conversely, in the uphill/downhill-walking and jogging groups, the medial meniscus extrusion at T2 was significantly higher than that at T1. Conversely, the medial meniscus extrusion at T3 was significantly lower than that at T2. By contrast, the lateral meniscus did not show any difference between the follow-up periods in any group.

**Conclusion:**

Temporary extrusion of the meniscus occurred after uphill/downhill tasks in healthy volunteers, and its reaction was observed only in the medial meniscus.

## 1 Introduction

Cartilage homeostasis maintains the appropriate balance between mechanical and metabolic stresses in healthy individuals. However, taxing activities, including sports, can cause extreme mechanical stress and distraction of homeostasis (1, 2). Cumulative mechanical stress is believed to affect structural changes in the knee compartment, resulting in knee injuries and overwork lesions, eventually leading to the onset of knee osteoarthritis (OA) (3, 4). Therefore, the detection of abnormal cumulative mechanical stress at an early stage is required to appropriately manage the prevention of lesions in the knee.

The meniscus absorbs shock and protects the knee cartilage (5). However, meniscal extrusion associated with dysfunction leads to the aggravation of the cartilage, resulting in knee pain and OA progression (6–8); therefore, it is recognized as the target factor for clinicians. By contrast, even healthy volunteers show temporary meniscus extrusion after marathons, which correlates with the amount of cumulative mechanical stress (9, 10). Furthermore, meniscus extrusion is observed to be highly sensitive to the cumulative stresses, including the repetition steps, rather than the peak value of mechanical stress (10). Therefore, meniscal extrusion can detect cumulative mechanical stress and is an indicator for the individual knee status.

However, the level of mechanical stress varies depending on environmental factors, including flat and uphill/downhill conditions (11, 12), and it is speculated that the cumulative stress can cause the reaction of the meniscus. Moreover, these stresses affect not only the medial compartment, but also the lateral side (13). However, only a few studies have reported the effects of different environmental factors on lateral and medial meniscus extrusion (MME). Addressing this knowledge gap could help establish appropriate management strategies for individual knees, potentially prolonging the duration of healthy athletic performance by preventing certain injuries. This study aimed to investigate the effects of cumulative uphill/downhill stress on the meniscal extrusion in each compartment. It was hypothesized that both menisci presented more extrusion in the uphill/downhill conditions.

## 2 Material and methods

### 2.1 Participants

Altogether 30 healthy volunteers with 30 asymptomatic knees were involved in this study [mean age, 22.0 ± 1.1 years; body mass index (BMI), 21.3 ± 2.1; men, *n* = 14]. The index knee was selected randomly for each participant. The participants included both runners and those engaged in recreational sport activities once a week as a habit, with no history of leg pain at least for 6 months. By contrast, participants were excluded based on the following criteria: (1) history of knee injury and surgical interventions, (2) chronic pain, and (3) experience of sports activity on the previous day. Hence, it is necessary to control the factors that affect the impact of participants’ performance and reaction to the meniscus. The demographic data are provided in Table 1.

**Table 1:** Participant characteristics.

	Flat-walking	Uphill/downhill-walking	Uphill/downhill-jogging	*p*-value
N/knees	10/10	10/10	10/10	
Gender (M:F)	4:6	4:6	6:4	
Age (years)	22.2 ± 0.4	21.8 ± 1.0	22.4 ± 1.1	1.02
Height (cm)	166.0 ± 9.4	165.7 ± 8.8	165.7 ± 4.8	1.0
Weight (kg)	57.2 ± 7.0	57.2 ± 7.0	61.0 ± 5.8	0.386
BMI (kg/m^2^)	21.7 ± 2.5	20.8 ± 2.0	22.2 ± 2.0	0.256

The values represent *t* mean ± standard deviation. *p*-value shows the significant differences among groups.

### 2.2 Study protocol and assessment

The evaluation was based on follow-up tests performed three times, including pre-effort (T1), post-effort (T2), and one day after effort (T3). This study followed an established protocol based on a previous study on the effects of mechanical stress on meniscal extrusion (10). Moreover, the effort time was observed between T1 and T2. The participants were instructed to avoid exercises such as sports and training that would affect the meniscus conditions during the study.

### 2.3 Efforts environment and classification group

All participants wore the same type of shoe (ME-WE432, Boston, MA, USA), according to their foot size, to minimize biases from the shoe properties on mechanical stress. The tasks were selected randomly by walking on a flat or uphill/downhill course, or jogging on an uphill/downhill course, and the participants were divided into flat-walking, uphill/downhill-walking, and uphill/downhill-jogging groups. In particular, the uphill and downhill terrain has an altitude of 70 m. The tasks covered a distance of 5 km and were performed at comfortable speeds.

### 2.4 Activity levels during task

During some tasks, activity levels were measured using specialized watches (ForeAthlete 55, Taipei, Taiwan). This device was wrapped around each participant's wrist and recorded data, including steps, pitch, and length during the effort. Subjective fatigue was immediately evaluated using the visual analog scale (VAS) after efforts. These parameters were compared among the different types of efforts.

### 2.5 Evaluation of meniscus extrusion

**Figure 1:**
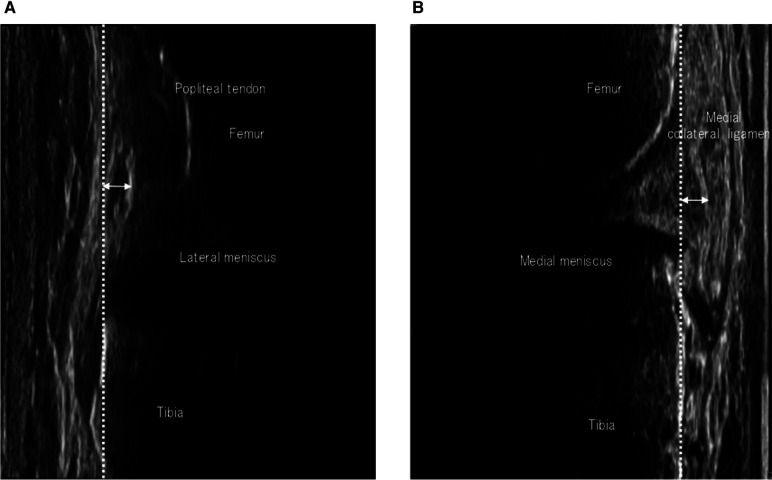
Images of meniscus extrusion. Coronal images of the lateral (**A**) and medial menisci (**B**). The reference lines according to the tibial plate and the distance of the meniscal extrusion are shown as dashed and arrowed lines, respectively.

The meniscus was evaluated using ultrasonography (SNiBLE, KONICA MINOLTA, Japan) with a prototype 3–11 MHz special linear-array transducer. T2 was performed immediately after the participants returned from the load course to our lab. During the measurement, the longitudinal transducer was placed on both medial and lateral joint spaces, and the triangular meniscus appeared as an echogenic structure between the femoral condyle and the tibial plateau, respectively. Appropriate images were determined based on specific landmarks, and the medial and lateral sides were determined as clear visualizations of the medial collateral ligament or origin of the popliteal tendon, based on a previous study (14, 15) (Figure 1).

The transducer was fixed using a flexible band that enabled comfortable walking. The video mode of the ultrasonography with a sampling rate of 30 Hz was recorded during walking, and an image of the meniscus was obtained. This ultrasonographic measurement was performed simultaneously with a wearable sensor using the outside signal of the device. Kinovea software (v0.8.15; Kinovea open-source project, www.kinovea.org) was used to adjust the match to the required sampling frequency.

**Figure 2:**
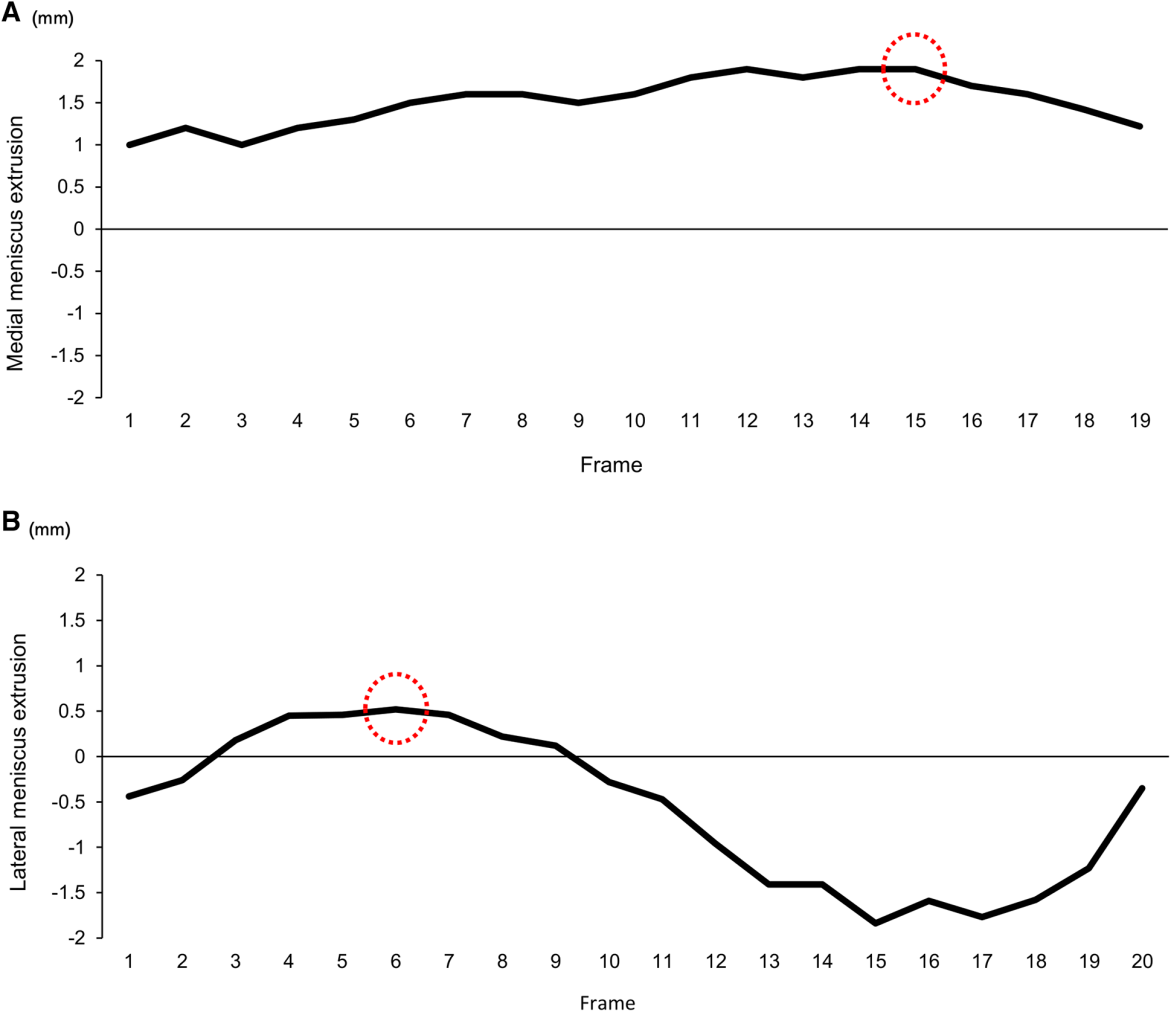
The waveform of meniscus extrusion during walking. A representative image of the waveform on the extrusion meniscus during walking shows the (**A**) medial and (**B**) lateral side. These waveforms were structured based on the value of the meniscus extrusion, obtaining approximately 20 continuous images. The red dot circle shows the point of the maximum value of meniscal extrusion.

The meniscus extrusion was defined as the quantitative value of expansion to the line of the tibial cortex. This process obtained approximately 20 images in a single trial and generated the waveform of the meniscal extrusion using the continual values of the extrusion of the meniscus. The maximum point was detected and used as a representative value for meniscal extrusion (Figure 2). These methods have been established as having high reliability in previous studies (14, 15). Moreover, the values were calculated as Δ-value based on the difference in meniscus extrusion between (T1) and (T2).

### 2.6 Assessments of gait form

**Figure 3:**
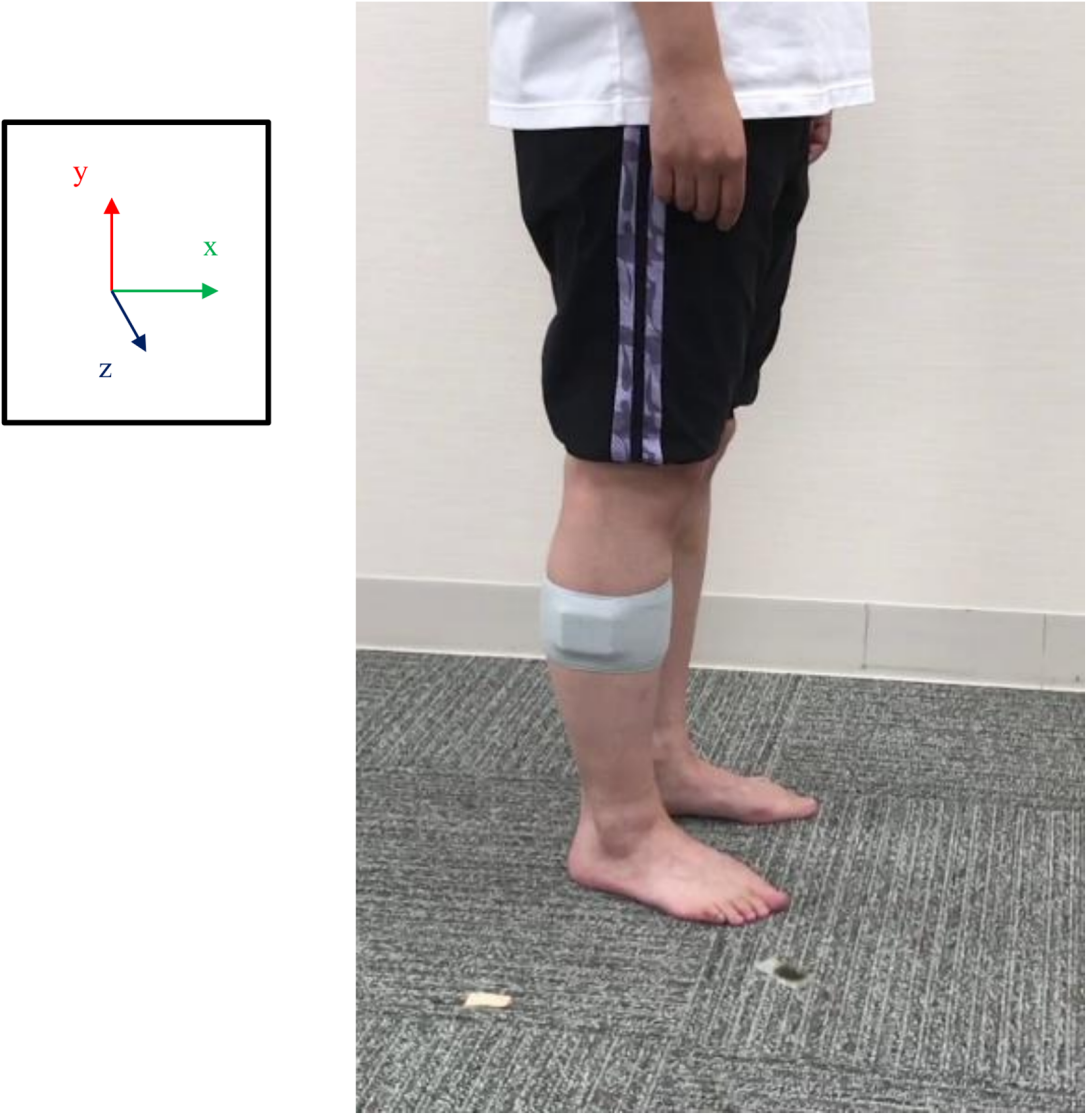
The location of the attached acceleration sensors. A view of the acceleration sensor attached to the shank is presented. The sensors were carefully attached to the shank on the longitudinal axis, with the midpoint between the lateral tibial plateau and the head of the fibula. The origin of the sensors was the *y*-axis oriented in the vertical direction and the *x*- and *z*-axes in the anteroposterior and mediolateral directions, respectively.

In this study, a single wearable sensor including an acceleration sensor and gyroscope with rate of 100 Hz (WAA-010, ATR-Promotions, Japan) evaluated the participant's gait form. The sensor was fixed to the midpoint between the lateral tibial plateau and the head of the fibula using a belt. In particular, the sensors were carefully attached to the shank longitudinally. Joint axes showed *y*-axis orientation in the vertical direction and *x*- and *z*-axes in the anteroposterior and mediolateral directions (Figure 3).

The participants were instructed to walk along a 10-m walkway three times. The analysis interval was set in the single-stance phase of a gait cycle and did not include gait initiation or termination. The gait event of the single-stance phase from heel contact to foot-off was estimated using the mediolateral angular velocity negative peaks, following a previous study (16). The video data were also obtained simultaneously and visually confirmed the gait events to obtain the minimum measurement error. During the analysis, raw data were filtered through a low-pass until reaching a 10 Hz cut-off, and peak values that can estimate gait events were detected using MATLAB software (MATLAB 2015a, MathWorks, Japan). Moreover, the perturbation of the shank accelerations in each direction during the stance phase was represented as root mean square (RMS), and a high value shows greater perturbation during walking. These protocols were based on a pilot study to confirm intraclass correlation coefficients (ICC), in which the RMS value was compared between test and re-test in six participants (x_ICC: 0.83, y_ICC: 0.9, z_ICC: 0.81).

### 2.7 Statistical analysis

Shapiro–Wilk test was used to confirm the data with or without normality. Within the group or follow-ups, VAS score, meniscus data, and walking parameters including activity and shank RMS were analyzed by multiple comparisons with the Bonferroni correction or Dunn test. The software Statistical Package for the Social Sciences (SPSS) (v23, IBM, Japan) was used to determine the critical *p*-value for statistical significance (*p* < 0.05).

Moreover, power analyses were performed as a *post-hoc* test to confirm the hypothesis using G-power. The differences in MME were compared between T1 and T2 in the uphill/downhill condition, and the effect size and power were 1.51 and 0.98, and 0.94 and 0.75 in the walking and jogging task, respectively.

## 3 Results

### 3.1 Characteristics of participants and activity in the efforts

The demographic data are provided in Table 1. No significant differences were observed between the groups. The average number of steps and the duration of effort in the flat and uphill/downhill-walking groups were significantly higher than that in uphill/downhill-jogging group. However, the pitch in uphill/downhill-jogging group was significantly higher than those in the flat and uphill/downhill-walking groups (Table 2).

**Table 2:** The comparisons among activities during efforts.

	Flat-walking	Uphill/downhill- walking	Uphill/downhill- jogging
Time (min)	60.1 ± 5.0^a^	68.8 ± 10.8^a^	36.3 ± 6.2
Step (step)	7,170.5 ± 525.3^a^	7,950.2 ± 949.9^a^	5,929.0 ± 669.1
Length (km)	5.1 ± 0.1	5.4 ± 0.3	5.3 ± 0.2
Pitch (step/min)	108.0 ± 10.5	94.6 ± 17.1	149.9 ± 12.4^b^

Pitch shows the average during effort. The values represent the mean ± standard.

^a^ Significantly higher than that in uphill/downhill-jogging (*p* < 0.05).

^b^ Significantly higher than that in walking and uphill/downhill-walking (*p* < 0.05).

### 3.2 Effect of load stress on the meniscus extrusion in mediolateral compartments

**Figure 4:**
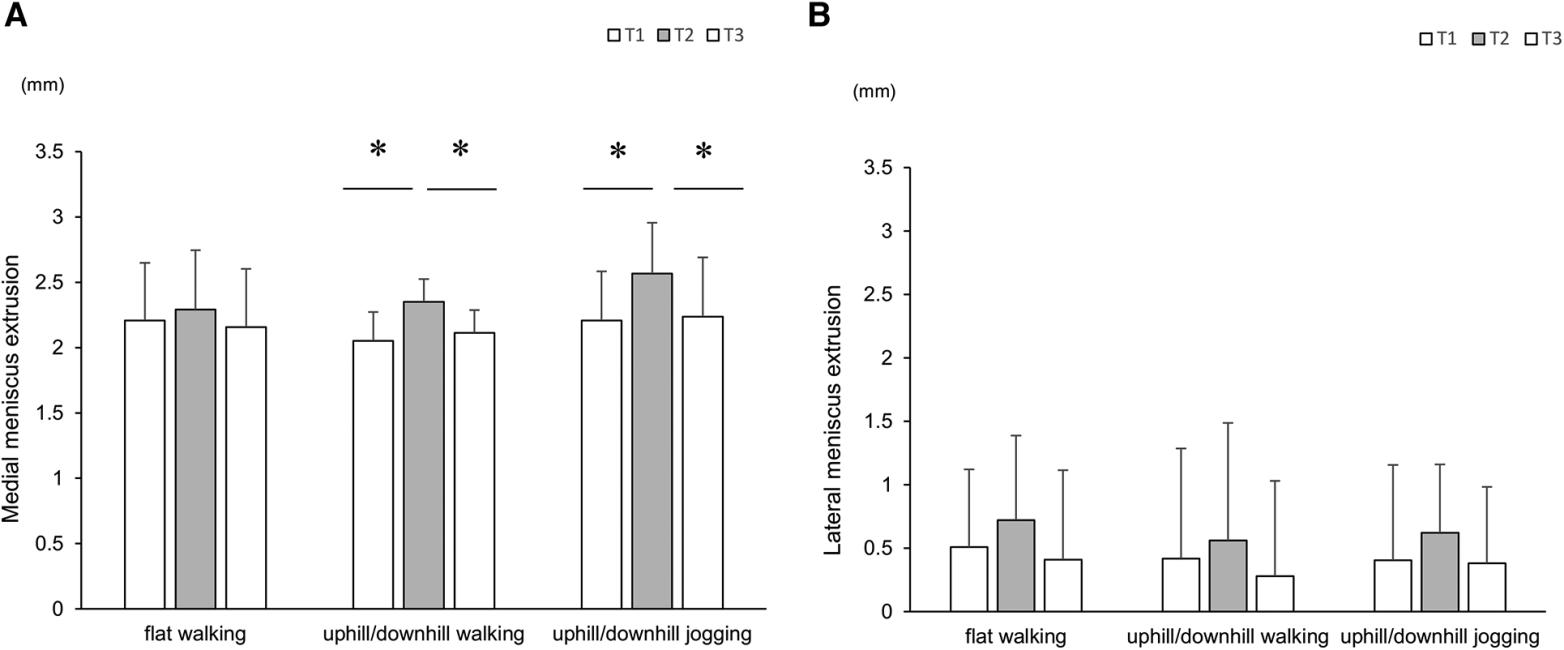
Comparisons of meniscus extrusion with time course. Plots showing the medial (**A**) and lateral menisci (**B**) These values are expressed as mean ± standard deviation. * indicates a significant difference between follow-up visits (*p* < 0.05).

In the uphill/downhill-walking and jogging groups, the MME at T2 was significantly higher than that at T1. Conversely, the MME at T3 was significantly lower than that at T2 (Figure 4A). The Δ-values in the uphill/downhill-walking and jogging were significantly higher than those in flat-waking (flat-walking: 0.1 ± 0.1, uphill/downhill-walking: 0.3 ± 0.2, uphill/downhill-jogging: 0.4 ± 0.3; flat-walking–uphill/downhill-walking: *r* = 1.52, flat-walking–uphill/downhill-jogging: *r* = 1.39, uphill/downhill-walking–uphill/downhill-jogging: *r* = 0.29).

In all groups, the lateral meniscus extrusions at T2 were higher than those at T1, although there were no significant differences (Figure 4B). Moreover, the Δ-value was not statistically significant among groups (flat-walking: 0.2 ± 0.3, uphill/downhill-walking: 0.1 ± 0.5, uphill/downhill-jogging: 0.2 ± 0.8).

### 3.3 Gait parameters based on wearable sensors

On the medial side, in the flat-walking group, the RMS of the anteroposterior direction was significantly higher at T2 than at T1, and the reaction remained at T3. Moreover, the RMS of the mediolateral direction was significantly higher at T3 when compared with that in T2. In the uphill/downhill-walking and jogging groups, the RMS values of the anteroposterior directions were significantly higher at T3 than those at T2 (Table 3).

**Table 3:** Correlation of gait form follow-up.

	Medial side			Lateral side		
	T1	T2	T3	T1	T2	T3
Flat-walking						
Shank *x* RMS	3.4 ± 0.6	4.2 ± 0.8^a^	4.2 ± 0.9^a^	3.2 ± 0.5	4.2 ± 1.0^a^	3.9 ± 1.5
Shank *y* RMS	2.8 ± 0.5	3.1 ± 0.7	2.9 ± 0.9	2.8 ± 0.6	3.2 ± 0.7	2.8 ± 0.9
Shank *z* RMS	2.2 ± 0.6	2.5 ± 0.7	2.7 ± 0.6^a^	2.2 ± 0.7	2.8 ± 0.9^a^	2.6 ± 1.0
Uphill/downhill-walking						
Shank *x* RMS	3.7 ± 1.2	3.9 ± 1.3	4.4 ± 1.0^a^	3.6 ± 1.3	4.2 ± 1.0	4.2 ± 1.2
Shank *y* RMS	2.8 ± 0.5	2.7 ± 0.5	3.0 ± 0.5	3.0 ± 0.9	2.8 ± 0.4	3.1 ± 0.5
Shank *z* RMS	2.4 ± 0.7	2.6 ± 0.7	2.7 ± 0.7	2.4 ± 0.9	2.7 ± 0.8	3.0 ± 0.9
Uphill/downhill-jogging						
Shank *x* RMS	3.8 ± 0.4	4.2 ± 0.7	4.7 ± 0.9^a^	4.5 ± 1.1	4.4 ± 0.6	4.9 ± 1.2
Shank *y* RMS	2.9 ± 0.7	3.0 ± 0.7	2.9 ± 0.8	3.2 ± 0.9	3.1 ± 0.8	3.0 ± 0.9
Shank *z* RMS	2.6 ± 0.4	2.9 ± 0.6	2.8 ± 0.6	2.7 ± 0.6	3.1 ± 0.7	3.1 ± 0.8

*y*-axis oriented in a vertical direction, *x*- and *z*-axes in anteroposterior and mediolateral directions. Pitch shows the average during effort. The values represent the mean ± SD.

^a^ Significantly higher than that in T1 (*p* < 0.05).

On the lateral side, in the flat-walking group, the RMS values in the anteroposterior and mediolateral directions were significantly higher at T2 than at T1 (Table 3).

In all groups, the stance time of the gait cycle was not significantly different between the follow-ups. Moreover, RMS and stance time had no significant correlation with Δ-value on both the medial and lateral sides.

### 3.4 The subjective assessment after efforts

The values of the VAS were 32.1 ± 24.7 mm, 36.1 ± 16.8 mm, and 79.1 ± 9.2 mm in the flat-walking and the uphill/downhill-walking and jogging groups, respectively. The VAS scores in the uphill/downhill-jogging group were higher than those in the flat and uphill/downhill-walking groups. However, there was no correlation with Δ-value in any group.

## 4 Discussion

The findings showed the effect of the uphill/downhill effort on the meniscus and temporary increase in meniscal extrusion, especially on the medial meniscus.

In this study, the participants performed tasks of the same length but with different time durations, steps, and pitches. However, these features were not directly related to meniscal reactions that revealed extrusion only in the uphill/downhill effort. Therefore, the uphill/downward motion itself could be a factor that affects meniscal extrusion, which partially supports the hypothesis. Greater meniscal extrusion correlates with concentrated mechanical stress in the knee compartment (8, 14, 17), and a previous study demonstrated a correlation between the amount of cumulative moment and aggravation of meniscal extrusion (10). In particular, the knee joint is strongly affected by the environment in which a high moment occurs during walking in uphill/downhill conditions rather than flat conditions (11, 12). These previous studies explain the finding that meniscal extrusion temporarily expands with greater mechanical stress.

The reaction of the meniscus on the knee compartment was observed on the medial side but not on the lateral side. A previous study investigated the amount of loading stress in each knee compartment and demonstrated that it occupied the medial compartment superiorly during walking or downhill-walking (12). Thus, it can be concluded that walking and uphill/downhill tasks mainly involve loading stress on the medial compartment and predominantly work on the medial meniscus. By contrast, the lateral meniscus shows a physiological intrusion within the tibial plateau (18) and the dynamics of drawing to the tibial plateau during walking have been observed (15). Gokkus et al. reported the relationship between the amount of meniscal extrusion and the stress distribution pattern of the meniscus using finite-element analysis. Their results showed that the meniscus experienced high mechanical stress depending on the location of the extrusion on the tibial plateau (19). In this study, the lateral meniscus showed similar dynamics and had a small extrusion value compared with the medial meniscus. Therefore, it can be concluded that the lateral meniscus may experience insufficient mechanical stress to detect changes in its location. Based on these results and those of previous studies, the medial meniscus could be concluded to serve as an indicator to reflect the cumulative mechanical stress in a knee joint.

In the present study, meniscal extrusion was caused by walking. Unfortunately, the RMS in the anterior–posterior direction was greater even after efforts according to the reaction of the MME. Thus, concerns may be raised that the change in gait form affects the meniscal extrusion rather than the cumulative mechanical stress. In the extrusion of the medial meniscus during walking, a previous study showed a significant correlation with the peak value of the knee vertical direction on the ground reaction force in healthy volunteers (14). In this study, the RMS in the vertical direction, which is associated with the ground reaction force, did not change during follow-up. Moreover, the RMS did not significantly correlate with the MME. A previous study and its findings suggest that the changes in gait form itself did not directly affect MME.

The subjective assessment did not show a significant difference between the flat and uphill/downhill-walking tasks, whereas the MME was only increased after the uphill/downhill-walking. Thus, exercise intensity obtained as a subjective assessment could not be adopted to prevent knee lesions associated with greater mechanical stress. In young athletes, medial meniscal extrusion is present and is associated with joint abnormalities such as meniscal tears and effusion; however, they do not suffer from knee pain and have no history of knee problems (20).Hence, based on several studies, a greater MME is known to be a risk factor for cartilage lesions and knee OA (7, 21). These studies support the fact that overload or diseases or injuries occur unconsciously. Thus, the findings and those of previous studies emphasize that it is necessary not only to provide information on the subjective score but also to objectively evaluate the control of mechanical stress to prevent knee lesions.

Typically, abnormal meniscus extrusion has been defined using various thresholds. Previous studies reported thresholds of 2, 3, or 4 mm under different situations, conditions, and patient populations (22–24), and did not reach a consensus on the appropriate value. By contrast, this study investigated the effect of cumulative mechanical stress on meniscus extrusion and quantified the extrusion without relying on a threshold. This method has been reported to have high reliability (8) and can detect the immediate reaction of meniscus extrusion after loading stress as the Δ-value. Therefore, this quantified evaluation could be a valuable tool for assessing the mechanical stress associated with meniscus extrusion.

This study has some limitations. First, this study had a small sample size; therefore, within-group analyses could not be performed. Second, the meniscus extrusion in the running group was measured during walking because of the low sampling rate on ultrasonography. This raises concerns about potentially underestimating the maximum value of meniscus extrusion after cumulative stress in the jogging condition. Third, this study did not obtain knee alignment. Finally, this study recruited healthy volunteers who engaged in recreational sports once a week. The athletes’ cumulative stress may not have been sufficiently reflected, and the distance was only 5 km. However, it is unknown whether these data can be applied. Future studies are needed to assess knee alignment at different effort distances with sufficient sample sizes.

## 5 Conclusion

Medial meniscus extrusion occurs temporarily after uphill/downhill efforts in healthy volunteers.

## Data Availability

The raw data supporting the conclusions of this article will be made available by the authors, without undue reservation.
